# Robot assisted radical nephrectomy with inferior vena cava thrombectomy; feasibility and outcome for level 3 thrombus with suprahepatic clamping

**DOI:** 10.1080/20905998.2026.2618296

**Published:** 2026-01-18

**Authors:** Kishore Thekke Adiyat, Atanu Kumar Pal, Jeni Mathew, Biju Chandran

**Affiliations:** a Department of Urology, Aster Medcity, Kochi, India; b Department of Gastrointestinal and HPB Surgery, Aster Medcity, Kochi, India

**Keywords:** Inferior vena cava thrombectomy, renal cell carcinoma, robotic surgery, suprahepatic clamping

## Abstract

**Objective:**

To assess the feasibility and safety of robot assisted radical nephrectomy with inferior vena cava thrombectomy (RARN-IVCT) in patients with Level 3 thrombus with suprahepatic clamping.

**Methods:**

This is a retrospective analysis of the patients who underwent RARN-IVCT for locally advanced renal cell carcinoma over 6 years. The perioperative, post operative and oncological outcomes were compared between the patients who underwent level 2 vs. level 3 RARN-IVCT.

**Results:**

A total of 27 patients who underwent RARN-IVCT during the study period were included in this study. Level I, II, III and IV thrombectomy were performed in two, 14, 10 and one patients, respectively. The mean duration of follow-up was 31.6 ± 20.5 months. The thrombus length, console time, IVC clamp time, total operating time, blood loss and hospital stay were significantly less in the patients with level II IVC thrombus (7.23 ± 2.75 vs. 9.69 ± 2.82 cm, 166.78 ± 90.54 vs. 325.75 ± 105.4 min, 20.84 ± 11.5 vs. 29.6 ± 8.42 min, 337.69 ± 135.1 vs. 529.56 ± 69.45 min, 631.47 ± 321.5 vs. 980.52 ± 453.62 ml, 5.7 ± 2.7 vs. 8.34 ± 3.2 days, level II vs. level III, respectively). The tumour size, the fall of serum creatinine and the complication rates were similar. Only six patients (three patients each in level II and level III) received blood transfusion.

**Conclusion:**

RARN-IVCT with supra-hepatic clamping can be used as a safe and feasible approach in carefully selected patients. It can provide good perioperative outcomes without increasing the complications and risking survival.

## Introduction

Radical nephrectomy (RN) with inferior vena cava (IVC) thrombectomy (RARN-IVCT) remains the gold standard treatment for locally advanced renal cell carcinoma (RCC) with IVC tumor thrombus [1]. IVC involvement is observed in approximately 4–10% of RCC cases and is associated with a poorer prognosis [1]. In appropriately selected patients – those with good performance status and no systemic metastasis – radical surgery offers a potential cure, with cancer-specific 5-year survival rates ranging from 40% to 65% [2]. Despite its curative intent, the procedure carries a perioperative mortality rate of 2–13%, according to a recent analysis by Bentin et al. [3].

Before the 1970s, surgical intervention for RCC with IVC thrombus was rare due to high complication rates and poor outcomes. The first formal report of IVC thrombectomy for RCC was published by Skinner et al. at Massachusetts General Hospital in 1972 [4]. Laparoscopic approaches to IVC thrombectomy were first explored in porcine models [5], with a few case reports between 2006 and 2010 describing pure laparoscopic and hand-assisted techniques – although these were largely limited to level II thrombi extending no more than 3 cm beyond the renal vein ostium [6]. In 2011, Abaza et al. reported the first series of robotic-assisted radical nephrectomy with IVC thrombectomy (RARN-IVCT) in six patients, demonstrating excellent outcomes with no complications, transfusions, or readmissions [7]. The robotic approach, offering enhanced dexterity, tremor reduction, wristed instruments, and high-definition three-dimensional visualization, has since enabled the safe execution of increasingly complex thrombectomies. Multiple studies have reported reduced blood loss, lower transfusion rates, shorter hospital stays, and fewer complications compared to open surgery [8].

Robotic approaches have now been extended to level III and, more recently, level IV IVC thrombi with encouraging results [9]. At our center, RARN-IVCT for level II thrombi was first performed in 2015. In 2021, we successfully performed our first level III thrombectomy with suprahepatic clamping. We subsequently evaluated the feasibility and safety of this technique for managing level III thrombi with suprahepatic IVC involvement.

## Materials and methods

This is a retrospective analysis of the patients who underwent robotic radical nephrectomy with IVC thrombectomy (RARN-IVCT) for locally advanced RCC over 6 years.

### Surgery

The da Vinci® Si (Intuitive Surgical, Sunnyvale, CA, USA) system was used for 22 cases. The other five cases were performed in the da Vinci® X and Xi system.

### Radical nephrectomy

For right-sided tumours, left-lateral decubitus position was used for the right-sided tumours. The renal artery was ligated in the inter-aortocaval area. Right Renal vein is stapled across and specimen extracted through a Pfannensteil incision. The right lobe of the liver is mobilized in this position for level III thrombus by dividing the right triangular ligament (Figure 1) The left radical nephrectomy is performed in the right lateral decubitus position is performed. The renal vein bearing thrombus is stapled across close to the IVC. The specimen is extracted through a Pfannensteil incision. For level III thrombus, the left lobe of the liver is mobilized by dividing the left triangular ligament in this position. Then the patient position is changed to left lateral decubitus to perform the IVC thrombectomy.

**Figure 1.**
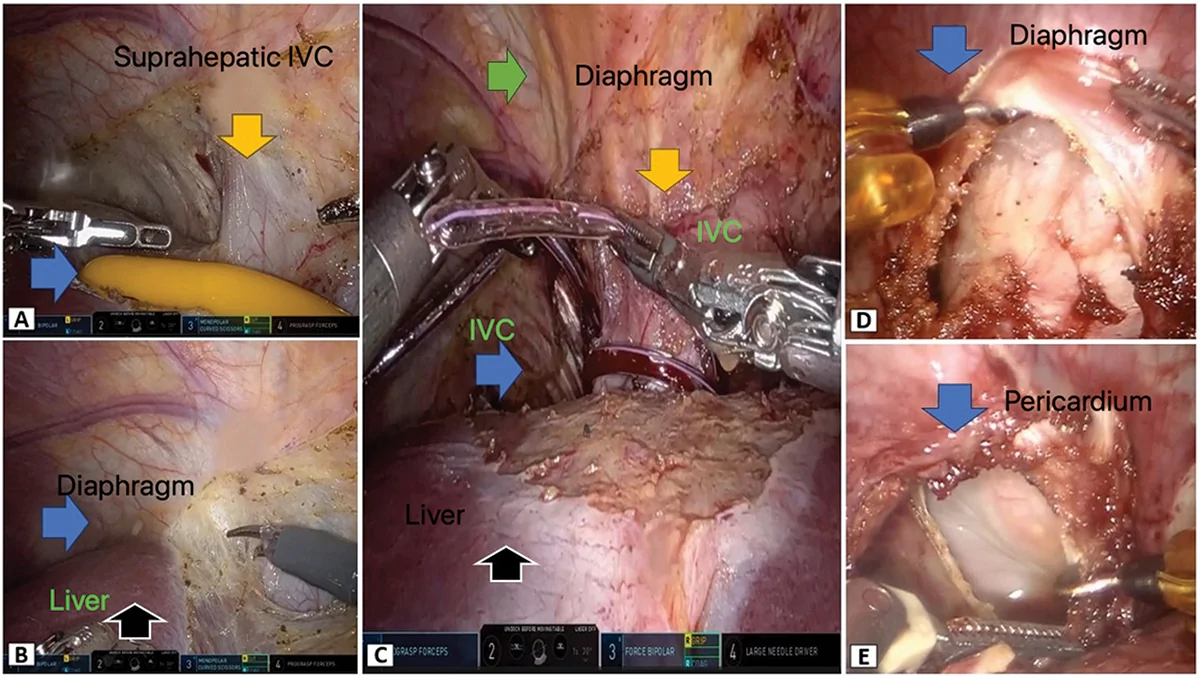
Suprahepatic IVC lengthening: (A) gold finger dissection of the liver (yellow arrow: suprahepatic IVC; blue arrow: gold finger dissector around the suprahepatic IVC); (B) circumferential dissection of IVC (blue arrow: Diaphragm; black arrow: Liver); (C) mobilization of the liver (yellow arrow: suprahepatic IVC; blue arrow: feeding tube around the suprahepatic IVC; green arrow: Diaphragm); (D) division of the central tendon of diaphragm (blue arrow: Diaphragm); (E) division of the pericardium (blue arrow: pericardium).

### Inferior venecaval dissection and liver mobilsation

A circumferential dissection of the infrahepatic IVC was performed along with the opposite renal vein for proper clamping. Meticulous dissection and division of all lumbar veins are performed. In the cases with level II thrombus which was extended into the intrahepatic portion, division of the short hepatic veins performed to get adequate exposure. For liver mobilsation and suprahepatic caval dissection, the patient position was made supine and subsequent mobilization proceeded in a clockwise direction from below. The dissection begins with the division of the ligamentum teres, and division of the falciform ligament. This incision is extended along the right superior coronary ligament, and continues through the left triangular ligament (Figure 1(A)). Next, the visceral peritoneum adjacent to the right side of the hepatic hilum and the infrahepatic vena cava is incised, along with division of the right inferior coronary and hepato-renal ligaments. The dissection is facilitated by retraction of the gallbladder using the robotic fourth arm, along with the use of a gold finger dissector introduced through the assistant port (Figure 1(A)). The liver is then completely mobilized off the IVC up to the hepatocaval ligament (Figure 1(B)). Small short hepatic and phrenic vein tributaries encountered during this dissection are clipped, while larger veins are controlled using Hem-o-lok clips or vascular staplers. An opening in the lesser omentum allows the control of porta hepatis looping it with a foley catheter (Figure 1(C)). The liver is meticulously dissected off the IVC until it rests in a piggyback position, remaining attached solely by the major hepatic veins. This manoeuvre allows for complete exposure of the infrahepatic and retrohepatic segments of the IVC. In addition to mobilizing the liver from the cava, developing a plane between the posterior surface of the IVC and the posterior abdominal wall is crucial. This step facilitates circumferential vascular control, which is essential should IVC resection or reconstruction become necessary.

Adequate suprahepatic lengthening is a critical step in all cases to facilitate safe, tension-free mobilization of the inferior vena cava (IVC). The extent of further dissection, including division of the central tendon and pericardium, is determined by the cranial extent of tumor involvement (Figure 1(D,E)). Suprahepatic mobilization is categorized into three levels: Suprahepatic infra-diaphragmatic, Suprahepatic extra-pericardial and Suprahepatic intra-pericardial. Final circumferential mobilization of the IVC is achieved from the left side to ensure complete 360-degree exposure. For vascular control, we prefer to use a 10 Fr infant feeding tube, which provides effective occlusion while minimizing trauma (Figure 1(C)).

### Thrombectomy

Sequential vascular control was achieved using vessel loops and Bulldog clamps on the caudal IVC, contralateral renal vein, porta hepatis, and suprahepatic IVC (Figure 2(A,B)). The IVC wall, including the stapler line, was then incised, and the thrombus was carefully extracted and placed in an endoscopic retrieval bag (Figure 2(C)). For level IV thrombus, milking the thrombus from the right atrium which was enabled using a real time transesophageal probe echo and robotic dropdown ultrasound probe, thrombectomy was accomplished. Following completion of the thrombectomy, intrahepatic control of the IVC was established, after which the clamps on the porta hepatis and suprahepatic IVC were released in a controlled manner. The IVC defect was closed using 5–0 Gore-Tex sutures, beginning from the superior and inferior corners to ensure a watertight closure. Vascular clamps were then sequentially released to restore circulation. Finally, the defect in the central tendon was closed using 3–0 V-Loc™ barbed sutures.

**Figure 2.**
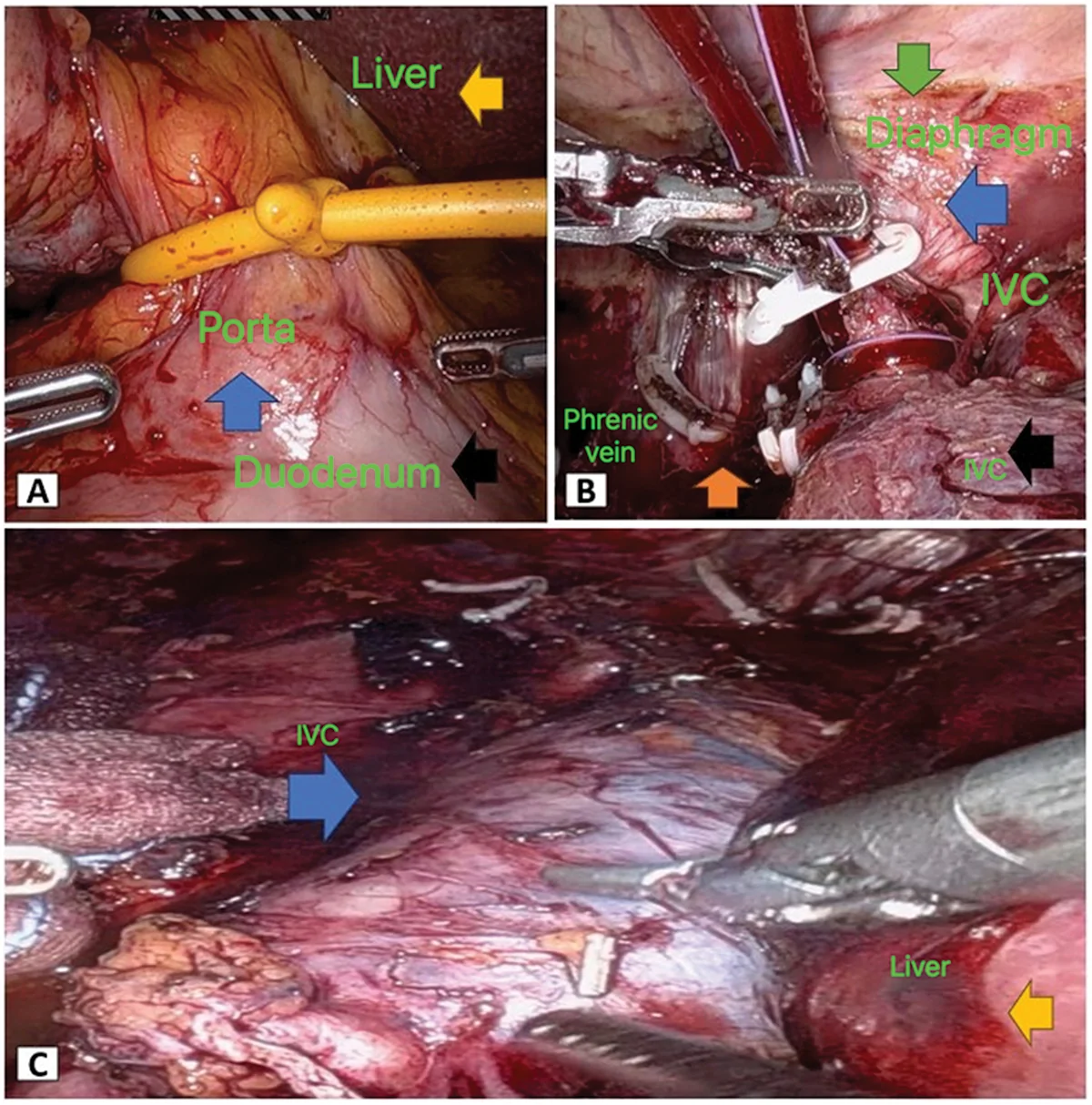
Level 3 robotic IVC thrombectomy: (A) control of porta hepatis (yellow arrow: undersurface of liver; blue arrow: hepatoduodenal ligament controlled with clamp; black arrow: Duodenum); (B) control of suprahepatic IVC (green arrow: Diaphragm; blue arrow: suprahepatic IVC control; black arrow: suprahepatic IVC containing thrombus; orange arrow: ligated inferior phrenic vein); (C) level 3 IVC thrombus (blue arrow: suprahepatic and retrohepatic IVC containing thrombus; yellow arrow: undersurface of liver).

### Parameters

Demographic data, comorbidities, laterality of the tumour, Eastern Cooperative Oncology Group (ECOG) performance status, renal function, tumour size and thrombus length as per the CT/MRI image, level of the thrombus, operative time, console time, IVC clamp time, blood loss, hemoglobin-drop, hospital stay, complication as per Clavien-Dindo grading, final histopathology and the follow up outcomes were evaluated. The follow-ups were done with clinical assessment, blood reports, chest X-rays, CT of the chest/abdomen fluorodeoxyglucose positron-emission tomography (FDG-PET). Those data were recorded.

### Statistical analysis

The statistical software utilized was IBM SPSS Version 27. Mean and standard deviation were used to express the data. Independent sample t-tests were performed for the continuous variables, while the Chi-square and Fisher’s exact tests were used for categorical data.

## Results

A total of 27 patients who underwent RARN-IVCT during the study period were included in this study. The mean age and BMI were 65.78 ± 8.5 years and 25.6 ± 4.8 kg/m2. Considering the level of IVC thrombus in our study, level I, II, III and IV thrombus were seen in two, 14, 10 and one patients, respectively (Table 1). The mean duration of follow-up was 31.6 ± 20.5 months. Three patients developed recurrence and metastasis. Five patients have died during the follow-up period (Table 1).

**Table 1.** Clinico-demographic and outcome data of the patients underwent robotic IVC thrombectomy.

	Parameters (*n* = 25)		Value
1	Age (years)		65.78 ± 8.5
	Sex (M/F)		25/2
2	BMI (kg/m2)		25.6 ± 4.8
3	Presenting Complains	Incidental	6
		Loss of appetite/weight	13/17
		Haematuria	16
		Dyspnoea	3
		Oedema	6
4	Laterality of Tumour	Right	19
		Left	8
5	ECOG	0	21
		1	6
6	H/o Pulmonary Embolism		5
7	Level of IVC Thrombus	1	2
		2	14
		3	10
		4	1
8	Complication	Wound infection	4
		UTI/Sepsis	2
		DVT	1
		Pulmonary Embolism	1
		AKI	3
9	Lymph node submitted/positive		3
10	Vein margin positivity		3
11	Adjuvant therapy given		4
12	T stage	T3a	2
		T3b	22
		T3c	3
13	Furham’s Grade	G2	10
		G3	11
		G4	6
14	Special feature	Sarcomatoid	4
		Rhabdoid	5
		LVI	14
15	Outcome	Disease-free	18
		Recurrence	4
		Deceased	5

Abbreviations: BMI: Body Mass Index; DM: diabetes Mellitus; HTN: Hypertension; CAD: coronary artery Disease; AKI: acute kidney Injury; CKD: chronic kidney Disease; ECOG: Eastern cooperative oncology group; UTI: urinary tract Infection; DVT: deep vein Thrombosis; LVI: lympho-vascular invasion.

We have assessed different perioperative parameters between the patients who had level 2 vs. level III thrombus who underwent robotic IVC thrombectomy. The thrombus length, console time, IVC clamp time, total operating time, blood loss and hospital stay were significantly less in the patients with level II IVC thrombus (7.23 ± 2.75 vs. 9.69 ± 2.82 cm, 166.78 ± 90.54 vs. 325.75 ± 105.4 min, 20.84 ± 11.5 vs. 29.6 ± 8.42 min, 337.69 ± 135.1 vs. 529.56 ± 69.45 min, 631.47 ± 321.5 vs. 980.52 ± 453.62 ml, 5.7 ± 2.7 vs. 8.34 ± 3.2 days, respectively). The tumour size, the fall of serum creatinine and the complication rates were similar. Only six patients (three patients each in level II and level III) received blood transfusion (Table 2).

**Table 2.** Comparison of different perioperative parameters between the patients who had level 2 vs. level 3 thrombus who underwent robotic IVC thrombectomy.

		Level 2 Thrombus (*n* = 14)	Level 3 Thrombus (*n* = 10)	*p*-value
1	Age	67.11 ± 9.27	64.53 ± 4.45	0.17
2	BMI	23.91 ± 4.55	25.78 ± 2.07	0.09
3	Tumour Size (cm)	9.5 ± 2.45	9.83 ± 2.63	0.38
4	Thrombus length (cm)	7.23 ± 2.75	9.69 ± 2.82	**0.001**
5	Console time (minutes)	166.78 ± 90.54	325.75 ± 105.4	**0.001**
6	Total IVC cross Clamp time (minutes)	20.84 ± 11.5	29.6 ± 8.42	**0.03**
7	Total operating time (minutes)	337.69 ± 135.1	529.56 ± 69.45	**0.0001**
8	Blood loss (ml)	631.47 ± 321.5	980.52 ± 453.62	**0.045**
9	Hemoglobin-drop (gm/dl)	1.85 ± 1.07	2.24 ± 0.2	0.09
10	Preop serum creatinine (mg/dl)	0.95 ± 0.78	1.24 ± 0.36	0.27
11	Postop serum creatinine (mg/dl)	1.98 ± 1.12	1.76 ± 0.64	0.19
12	Blood transfusion	3	3	0.65
13	Hospital-stay (days)	5.7 ± 2.7	8.34 ± 3.2	**0.02**
14	Complication Clavien-Dindo 2/3A	6/2	4/3	–

## Discussion

The Mayo Clinic classification is the most widely used system for categorizing IVC thrombi. Blute et al. introduced the now well-established concept of a level III thrombus, which extends to or above the hepatic veins but remains below the diaphragm [10]. In this article, we classified our patients according to the Mayo system. Through our experience, we observed that the key distinction between level II and level III thrombi lies in the need for suprahepatic versus infrahepatic caval control. In this discussion, we aim to highlight the technical nuances involved in the robotic approach to managing these two subsets of patients.

Despite the early success of robotic prostatectomy, it took over a decade before techniques for RARN-IVCT were formally described. The initial series by Abaza et al. laid the foundation for RARN-IVCT, with subsequent studies evaluating its feasibility, safety, and surgical outcomes [11]. In the ensuing years, there has been a gradual progression of RARN-IVCT from level I and culminating in addressing even level IV. Initial case series and reports mostly dealt with infrahepatic and intrahepatic IVC thrombus from 2011.

The technique for managing level II IVC thrombi has been standardized in previous publications [12]. The most critical components of level II RARN-IVCT include circumferential IVC dissection, accurate delineation of the thrombus extent both suprarenally and infrarenally to enable proximal and distal IVC control, ligation of lumbar veins to reduce bleeding, and isolation of the contralateral renal vein as part of the sequential clamping strategy. For IVC mobilization, the ‘minimal-touch’ technique described by Gill et al. is employed to prevent inadvertent thrombus embolization [13]. In this approach, pericaval tissue is gently retracted without direct manipulation of the IVC. The robotic platform offers improved access to the renal hilum, facilitating early ligation of the renal artery. However, in many cases – particularly in locally advanced RCC – the hilar anatomy may be complex due to aberrant vessels, newly formed high-caliber venous collaterals, and early multiple arterial branches. To address these challenges, Gill et al. developed the ‘midline-first, lateral-last’ approach, where in the interaortocaval region is dissected first to ligate the renal artery at its origin, avoiding manipulation of a potentially hostile hilum [13]. The 30-degree robotic camera enhances visualization, aiding in lumbar vein ligation and overall vascular control. Intraoperative tools such as the robotic ultrasound probe and TilePro™ technology are crucial for accurately determining the thrombus extent, detecting IVC wall invasion, and identifying any residual vascular flow after renal artery ligation [14]. Gill et al. and Chopra et al. recommend the use of an Endo-GIA vascular stapler to divide the renal vein containing thrombus, as it minimizes the risk of back-bleeding and embolization while facilitating safe removal of the IVC segment with thrombus [13,15]. Immediate bagging of the thrombus after extraction further helps prevent tumor spillage.

In the early years of RARN-IVCT, surgeries were predominantly limited to level II thrombi – those confined below the hepatic veins. Although Gill et al. successfully performed level IV thrombus removal under cardiopulmonary bypass, suprahepatic caval clamping was not attempted [16]. The primary challenge in managing level III IVC thrombi was the need for suprahepatic IVC control, which required extensive liver mobilization.

In 2015, de Castro Abreu et al. provided important anatomical insights through cadaveric studies, demonstrating that the suprahepatic segment of the IVC can be safely accessed and controlled using a purely robotic transperitoneal approach [17]. They were able to mobilize approximately 2–3 cm of the infradiaphragmatic suprahepatic IVC, which had an average diameter of 2.5 cm. Importantly, both the right and left hepatic veins were clearly visualized, supporting the feasibility of suprahepatic IVC dissection and control in a robotic setting – an essential step for managing higher-level thrombi. Building on this anatomical feasibility, Kundavaram et al. reported a novel endoluminal technique in 2016 for controlling the intrahepatic or retrohepatic IVC during robotic thrombectomy [12]. Their approach involved the use of an intracaval occlusion balloon inserted over a soft-tip guidewire, which was introduced into the IVC through a small venotomy made caudal to the right renal vein and cephalad to a Rummel tourniquet placed on the infrarenal IVC. The Fogarty catheter was advanced through the tumor thrombus to reach the desired intrahepatic location, allowing temporary occlusion of the cephalic IVC. This technique offered several advantages. Most notably, it enabled effective cephalad IVC control without extensive liver mobilization or wide IVC dissection. Additionally, thrombi with minimal adherence to the IVC wall could be extracted through a smaller cavotomy, thereby reducing operative time, blood loss, and technical complexity. However, recognizing the risk of thrombus migration during manipulation, the authors prudently pre-positioned a vessel loop around the suprarenal IVC as a precautionary measure. Building on previous endoluminal strategies, Alahmari et al. introduced a significant technical advancement by employing the right internal jugular vein as the access point for guidewire insertion [18]. This approach allowed for the deployment of a stent graft balloon catheter under fluoroscopic guidance, enabling precise and controlled occlusion of the cephalad IVC during robotic thrombectomy. By shifting the access site to the neck and utilizing imaging-guided positioning, this technique enhanced both procedural accuracy and safety, while minimizing manipulation of the retroperitoneal space.

In contrast, Zhang et al. explored a non-invasive alternative to cephalad IVC clamping in a prospective cohort study involving patients undergoing robotic level II–III thrombectomy [19]. Rather than relying on direct vascular occlusion, their method involved indirect IVC control by elevating the liver and transiently increasing intra-abdominal pressure to 20 mmHg via pneumoperitoneum. This elevated pressure effectively reduced venous return, mimicking the hemodynamic effects of traditional clamping without requiring vascular instrumentation. The findings were promising: the non-clamping group exhibited no cases of tumor thrombus dislodgement, gas embolism, or postoperative hypercapnia, affirming the safety and feasibility of this simplified approach. However, the technique is not without limitations. Maintaining a consistent pneumoperitoneum pressure can be technically demanding, and interpatient variability in venous compliance may lead to unpredictable bleeding risks. Nonetheless, the method offers a compelling, less invasive option in select patients, particularly when cephalad IVC exposure is technically challenging or contraindicated

In 2015, Hui et al. described an innovative thoracoscopy-assisted technique for accessing the suprahepatic inferior vena cava (IVC), laying the foundation for an entirely port-based approach to resect level III tumor thrombus [20]. Their method involved thoracoscopic hook electrocautery to create a longitudinal pericardial incision approximately 2 cm anterior to the phrenic nerve, performed under carbon dioxide insufflation with intermittent apnea to maintain optimal thoracic visualization. Diaphragmatic retraction was achieved using a laparoscopic finger retractor, improving inferior exposure of the IVC. To facilitate precise vascular dissection, an articulating, lighted curved dissector was used to isolate the suprahepatic IVC. Intraoperative real-time transesophageal echocardiography played a critical role in confirming complete cessation of caval flow following the application of a Rummel tourniquet. Although robotic thrombectomy of the right renal unit and IVC was attempted, the procedure was ultimately converted to open surgery due to dense adherence of the thrombus to the caval wall. Despite the need for conversion, this case represented a pivotal advancement in the pursuit of a fully robotic approach to level III IVC thrombi. The combined thoracoabdominal technique not only demonstrated the anatomical feasibility of suprahepatic IVC control via minimally invasive means but also highlighted key challenges, including tumor adherence and the need for advanced intraoperative imaging. This hybrid approach has since served as a conceptual springboard for the development of more refined and safer robotic techniques in high-level IVC thrombectomy.

We first described our technique for suprahepatic IVC dissection in 2022 [21]. Since then, we have applied this approach in 10 patients undergoing robotic-assisted level 3 IVC thrombectomy (Table 1). A key technical challenge in these cases was the robotic mobilization of the liver, which is essential for adequate exposure of the suprahepatic IVC. Our procedure begins with a nephrectomy performed in the lateral decubitus position. During this phase, we staple and retrieve the renal vein containing the tumor thrombus, thereby creating sufficient working space for subsequent liver mobilization (Figure 1B). In left-sided nephrectomies, the left lobe of the liver is mobilized concurrently; conversely, the right lobe is addressed during right-sided procedures. Following nephrectomy, the patient is repositioned to the supine position to facilitate further liver mobilization and control of the porta hepatis. We employ a ‘Goldfinger’ dissector to retract the liver atraumatically while ensuring adequate exposure (Figure 1A). In the supine position, we proceed with mobilization of the contralateral liver lobe, division of short hepatic veins, and dissection around the porta hepatis. Once both lobes have been mobilized, the suprahepatic IVC is readily visualized (Figure 1C).

For enhanced exposure, especially in Level III–IV thrombi, we adopted the technique described by Huang et al., which involves dividing the central tendon of the diaphragm to expose more of the infradiaphragmatic suprahepatic IVC [22] (Figure 2A). This manoeuvre proved especially beneficial in our first Level IV case, allowing for improved visualization and safer manipulation of the IVC [10]. We subsequently found it to be equally useful in Level III thrombi, as it consistently enabled the exposure of a longer segment of the suprahepatic IVC. Intraoperative use of transesophageal echocardiography and a drop-down robotic ultrasound probe was essential for real-time visualization of the thrombus and to guide precise dissection. With growing experience, liver mobilization has become increasingly efficient. In our series, porta hepatis clamp times remained within acceptable limits, and no instances of significant hepatic dysfunction were observed postoperatively. We prefer performing the thrombectomy in a modified supine or ‘right-side-up’ position (Figure 2). This orientation minimizes bowel interference, facilitates access to the right renal artery in left-sided tumors and retracts the right lobe of the liver away from the operative field, thereby improving visualization of the intrahepatic IVC. This positioning is especially advantageous in cases involving adherent thrombus or when partial IVC resection is anticipated. In one patient with level 3 IVC thrombus with previous surgical history of Whipple’s procedure, we used the described delayed occlusion of the proximal inferior vena cava (DOPI) technique which helped us to retrieve the thrombus without extensive suprahepatic dissection [23]. Overall, the robotic approach not only allows for meticulous dissection and precise vascular control but may also contribute to reduced intraoperative blood loss, decreased postoperative pain, and shorter hospital stay (Table 2).

In our study, we compared the perioperative outcomes between patients undergoing level II and level III RARN-IVCT. The idea behind was to assess the morbidity incurred while mobilising liver and clamping porta robotically. Patients with level II thrombus demonstrated significantly shorter thrombus length, console time, IVC clamp time, total operative duration, estimated blood loss, and hospital stay compared to those with level III thrombus (Table 2). However, there were no significant differences between the two groups in terms of hemoglobin drop, transfusion rates, pre- and postoperative serum creatinine levels, or overall complication rates (Table 2).

Despite the feasibility and safety of our approach, certain limitations must be acknowledged. One of the main drawbacks is the increased operative time associated with level III cases, largely due to the need of patient repositioning for multiple times – typically from lateral to supine – to facilitate different phases of the surgery. This repositioning not only extends the operative duration but also requires careful intraoperative coordination. An alternative approach has been described by Wang et al., involving mobilization of the suprahepatic IVC in the right lateral position specifically for right-sided tumors [24]. This technique may potentially eliminate the need for repositioning, thereby streamlining the procedure. Nonetheless, one persisting technical challenge – particularly in obese patients or those with hepatomegaly – is the handling and retraction of a bulky liver, which can impede exposure and increase operative complexity

## Conclusion

RARN-IVCT with supra-hepatic clamping for level 3 thrombus can be used as a safe and feasible approach. It can provide good perioperative outcomes without increasing the complications and risking survival.
